# Anti-Arthritic Effects of Magnolol in Human Interleukin 1β-Stimulated Fibroblast-Like Synoviocytes and in a Rat Arthritis Model

**DOI:** 10.1371/journal.pone.0031368

**Published:** 2012-02-16

**Authors:** Jyh-Horng Wang, Kao-Shang Shih, Jing-Ping Liou, Yi-Wen Wu, Anita Shin-Yuan Chang, Kang-Li Wang, Ching-Lin Tsai, Chia-Ron Yang

**Affiliations:** 1 Department of Orthopedic Surgery, National Taiwan University Hospital, Taipei, Taiwan; 2 Orthopedic Department, Shin Kong Wu Ho-Su Memorial Hospital, Taipei, Taiwan; 3 School of Pharmacy, Taipei Medical University, Taipei, Taiwan; 4 School of Pharmacy, College of Medicine, National Taiwan University, Taipei, Taiwan; Instituto de Biofisica Carlos Chagas Filho - Universidade Federal do Rio de Janeiro, Brazil

## Abstract

Fibroblast-like synoviocytes (FLS) play an important role in the pathologic processes of destructive arthritis by producing a number of catabolic cytokines and metalloproteinases (MMPs). The expression of these mediators is controlled at the transcriptional level. The purposes of this study were to evaluate the anti-arthritic effects of magnolol (5,5′-Diallyl-biphenyl-2,2′-diol), the major bioactive component of the bark of *Magnolia officinalis*, by examining its inhibitory effects on inflammatory mediator secretion and the NF-κB and AP-1 activation pathways and to investigate its therapeutic effects on the development of arthritis in a rat model. The *in vitro* anti-arthritic activity of magnolol was tested on interleukin (IL)-1β-stimulated FLS by measuring levels of IL-6, cyclooxygenase-2, prostaglandin E_2_, and matrix metalloproteinases (MMPs) by ELISA and RT-PCR. Further studies on how magnolol inhibits IL-1β-stimulated cytokine expression were performed using Western blots, reporter gene assay, electrophoretic mobility shift assay, and confocal microscope analysis. The *in vivo* anti-arthritic effects of magnolol were evaluated in a *Mycobacterium butyricum*-induced arthritis model in rats. Magnolol markedly inhibited IL-1β (10 ng/mL)-induced cytokine expression in a concentration-dependent manner (2.5–25 µg/mL). In clarifying the mechanisms involved, magnolol was found to inhibit the IL-1β-induced activation of the IKK/IκB/NF-κB and MAPKs pathways by suppressing the nuclear translocation and DNA binding activity of both transcription factors. In the animal model, magnolol (100 mg/kg) significantly inhibited paw swelling and reduced serum cytokine levels. Our results demonstrate that magnolol inhibits the development of arthritis, suggesting that it might provide a new therapeutic approach to inflammatory arthritis diseases.

## Introduction

Inflammatory arthritis is a synovial disease characterized by chronic inflammation of the joints and can result in disability owing to joint destruction. Proliferative fibroblast-like synoviocytes (FLSs) play crucial roles in both the propagation of inflammation and joint damage, as they produce large amounts of pro-inflammatory mediators, such as interleukin (IL)-1, IL-6, tumor necrosis factor-α (TNF-α), matrix metalloproteinases (MMPs), and prostaglandin E_2_ (PGE_2_) [1]. These mediators bind to specific receptors, causing gene transcription, and form complicated signaling interactions which contribute to the progression of inflammatory arthritis, e.g. leukocyte infiltration, cytokine networks formation, cartilage catabolism elevation and anabolism suppression [2]. Non-steroidal anti-inflammatory drugs (NSAIDs) are the principal treatment for arthritis patients; however, they only inhibit cyclooxygenases (COXs) and reduce prostaglandin generation and have no effect on the production of inflammatory cytokines [3]. In addition, anti-inflammatory agents carry the risk of gastrointestinal toxicity. A new generation of NSAIDs, such as rofecoxib, has been developed with the aim of avoiding adverse gastrointestinal effects, but rofecoxib had to be withdrawn from the market due to cardiovascular toxicity. Furthermore, although some biologic agents, such as etanercept and infliximab, have provided major advances in treatment, they are expensive and must be injected subcutaneously or intravenously, which, in turn, increases the risk of infection [4]. Thus, side-effects remain one of the problems of the long-term use of NSAIDs and there is a need for safe and effective anti-arthritic agents for long-term use. Recent study showed that piascledine, mixture of nonsaponifiable components of avocado and soybean oils, exerts promising effect to relief inflammatory arthritis symptoms [5]; several groups also have studied small anti-inflammatory molecules derived from natural sources [6], [7] with the aim of developing new treatments, but scientific evidence of their anti-arthritic efficacy is still insufficient.

The biphenyl neolignan magnolol (5,5′-Diallyl-biphenyl-2,2′-diol) is purified from the commonly used Chinese medicinal herb *Magnolia officinalis*, which has long been used for the treatment of fever, headache, asthma, anxiety, and diarrhea [8]. Since the NOAEL (no observed adverse effect level) of magnolol greater than 240 mg/kg body weight per day in a 90 day study in rats, it has been classified as no safety concern food additives by World Health Organization [9]. Magnolol has been shown to possess a range of pharmacological effects, including an anti-inflammatory effect [10], anti-thrombotic effect [11], anti-tumor effect [12], [13], a platelet aggregation inhibitory effect [11], [14], and anti-oxidant activity [15]. It has been shown to suppress the expression of inducible nitric oxide synthase [16] and COX-2 by macrophages [17], reduce the production of the atherosclerosis mediators monocyte chemotactic protein-1 and vascular cell adhesion molecule-1 by endothelial cells [18], and inhibit secretion of MMPs, IL-8, and TNF-α by different cell types [19], [20]. Interestingly, no severe side effects of magnolol have been reported. These effects of magnolol would seem to be beneficial for chronic inflammatory diseases, for example, inflammatory arthritis, but, to our knowledge, the anti-arthritic efficacy of magnolol has never been evaluated.

The present study was performed to examine the inhibitory effect of magnolol on the expression of pro-inflammatory cytokines and enzymes in IL-1β-stimulated FLS and to elucidate the underlying mechanisms. Magnolol was found to reduce IL-1β-induced IL-6, COX-2, MMP-1, and MMP-13 expression and these effects correlated with its inhibition of NF-κB and MAPK activation. Furthermore, studies using an adjuvant-induced arthritis rat model showed that magnolol inhibited the development of arthritis, suggesting its potential as a therapeutic agent in inflammatory arthritis.

## Results

### Magnolol suppresses inflammatory mediator production by IL-1β-stimulated FLS

**Figure 1:**
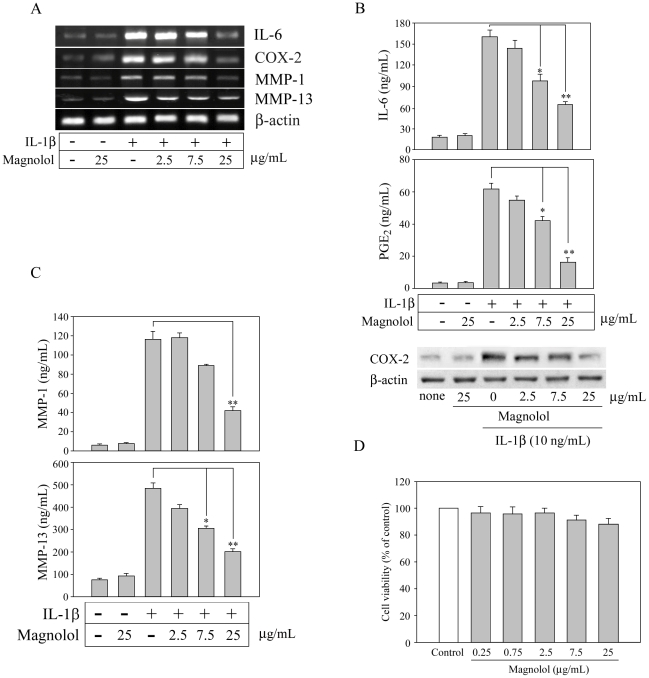
Magnolol inhibits the IL-1β-induced production of pro-inflammatory mediators in a concentration-dependent manner. (A) 1×10^6^ fibroblast-like synoviocytes (FLS) were treated with magnolol (0–25 µg/mL) for 30 min, then were incubated for 5 h with or without 10 ng/mL of IL-1β in the continued presence of magnolol, then levels of IL-6, COX-2, MMP-1, and MMP-13 mRNAs were measured by RT-PCR. (B, C) FLS were incubated with magnolol (0–25 µg/mL) for 30 min, then with IL-1β (10 ng/mL) for 24 h in the continued presence of magnolol, then supernatants were assayed for (B) IL-6 or PGE_2_ or (C) MMP-1 or MMP-13 by ELISA; and the whole cell extracts were subjected to Western blot analysis for COX-2 (B). (D) Viability of FLS determined after 24 h treatment with 0.25–25 µg/mL of magnolol compared to the control group using the MTT assay. In (B)–(D), the data are the mean ± standard error of the mean (SEM), with n = 3. * *p*<0.05 and ** *p*<0.01 compared to the indicated groups.

To examine the anti-inflammatory effect of magnolol, FLS were stimulated with 10 ng/mL of IL-1β in the presence or absence of magnolol. As shown in **figure 1**, addition of IL-1β not only significantly increased mRNA (**Figure 1A**) and protein (**Figure 1B and C**) levels of IL-6, COX-2, MMP-1, and MMP-13 (both overexpress in inflammatory arthritis cartilage and are rate-limiting components of the collagen degradation process), but also increased production of PGE_2_ protein (**Figure 1B**). Interestingly, magnolol pre-treatment markedly inhibited the IL-1β-induced increase in IL-6, COX-2, MMP-1, and MMP-13 mRNA and protein and PGE_2_ secretion in a concentration-dependent manner (**Figure 1A–C**). This inhibition was not due to decrease total protein levels, since β-actin levels were unchanged (**Figure 1B**) and none of the treatments had any significant effect on cell viability at 24 h, assessed using the MTT assay (**Figure 1D**).

### Magnolol inhibits NF-κB and MAPK signaling in IL-1β-activated FLS

**Figure 2:**
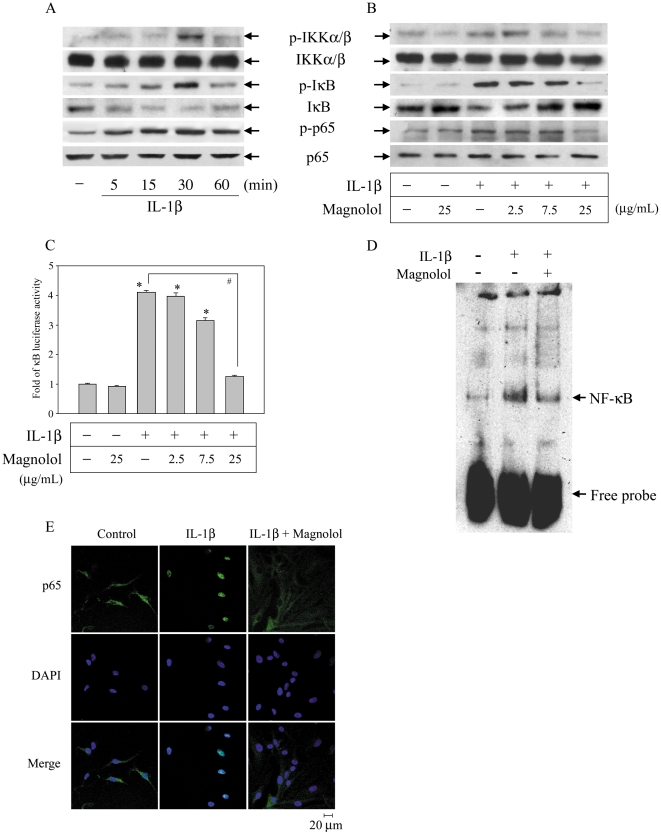
Magnolol inhibits NF-κB activation in IL-1β-stimulated FLS. FLS (1×10^6^ cells) were treated with or without 10 ng/mL of IL-1β for the indicated times (A) or were treated with 0–25 µg/mL of magnolol for 30 min, then stimulated with 10 ng/mL IL-1β for 30 min in the continued presence of magnolol (B), then the cells were harvested and whole cell extracts subjected to Western blot analysis for the indicated proteins. (C) Cells (1×10^5^ cells) were transiently transfected with 1 µg of pGL4.32[*luc2P/*NF-κB-RE*/*Hygro] for 24 h and treated with 0–25 µg/mL magnolol for 30 min prior to stimulation with 10 ng/mL of IL-1β in the continued presence of magnolol for a further 6 h, then luciferase activity was measured as described in the Materials and Methods. The results are expressed as the mean ± standard error of the mean (SEM), with n = 3. * *p*<0.05 compared to the control group; #*p*<0.05 for comparison of indicated groups. (D) FLS were incubated with vehicle or 10 ng/mL of IL-1β or were pretreated with 25 µg/mL of magnolol for 30 min, then incubated with IL-1β in the continued presence of magnolol for 1 h, then the DNA binding activity of the nuclear extracts was examined in an electrophoretic mobility shift assay using a specific NF-κB DNA probe. (E) Cells (1×10^5^ cells) were left untreated or were treated with 25 µg/mL of magnolol for 30 min, then stimulated with 10 ng/mL of IL-1β in the continued presence of magnolol for 1 h, when samples were prepared for confocal microscopy analysis. Scale bar = 20 µm.

**Figure 3:**
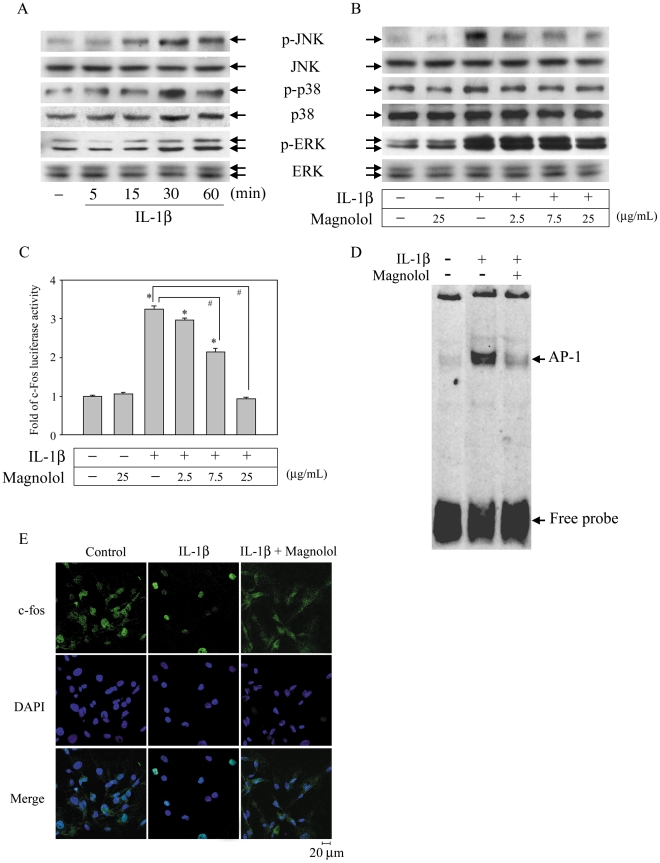
Magnolol inhibits MAPK pathways and c-fos nuclear translocation. Cells were treated with 10 ng/mL of IL-1β for different time periods (A) or were treated with magnolol at the indicated concentration for 30 min, then stimulated with IL-1β in the continued presence of magnolol for 30 min (B). The cells were then harvested and whole cell extracts prepared for Western blot analysis for the indicated proteins. (C) Cells (1×10^5^ cells) were transiently transfected with 1 µg of p5xATF6-GL3 for 24 h, then incubated with 0–25 µg/mL magnolol for 30 min before activation with 10 ng/mL of IL-1β in the continued presence of magnolol for another 6 h, when luciferase activity was measured as described in the “Materials and Methods”. The results are expressed as the mean ± standard error of the mean (SEM), with n = 3. * *p*<0.05 compared to the control group; #*p*<0.05 for the comparison of the indicated groups. (D) Cells were incubated with vehicle or IL-1β (10 ng/mL) for 1 h or were pretreated with 25 µg/mL of magnolol for 30 min, then stimulated with IL-1β in the continued presence of magnolol for 1 h. Nuclear extracts were then subjected to a DNA-binding reaction with oligonucleotides specific for AP-1. The specific DNA-binding activity of the AP-1 complex is indicated by an arrow. (E) Cells (1×10^5^ cells) were incubated with or without 25 µg/mL of magnolol for 30 min, then stimulated with 10 ng/mL of IL-1β in the continued presence of magnolol for 1 h, when samples were prepared for confocal microscopy analysis. Scale bar = 20 µm.

The NF-κB and MAPKs pathways play pivotal roles in the expression of inflammatory mediators by IL-1β-stimulated FLS and contribute to inflammatory arthritis [21], [22]. To further characterize the molecules involved in the inhibitory effect of magnolol, we examined whether magnolol regulated these signaling pathways. FLS were treated with magnolol (0–25 µg/mL) for 30 min prior to stimulation with 10 ng/mL of IL-1β in the continued presence of magnolol for different time periods, then levels of the phosphorylated or total forms of IKKα/β, IκBα, and p65 were measured using Western blotting. IL-1β treatment for 30 min not only caused significant phosphorylation of IKKα/β at serine 180/181, phosphorylation of IκBα at serine 32, and IκBα degradation, but also increased phosphorylation of p65 (**Figure 2A**). Interestingly, magnolol pre-treatment markedly inhibited all four effects in a concentration-dependent manner (**Figure 2B**). The result of a promoter activity assay showed that magnolol caused concentration-dependent inhibition of IL-1β-mediated NF-κB promoter activation (**Figure 2C**). Furthermore, after 1 h treatment with IL-1β, a significant increase in NF-κB-DNA binding activity was seen in an EMSA (**Figure 2D**) and a dramatic increase in the translocation of NF-κB into the nucleus was observed by laser confocal microscopy (**Figure 2E**) and both effects were markedly inhibited by addition of magnolol (**Figure 2D** and **E**). IL-1β treatment also resulted in a significant increase in phosphorylation of JNK, p38, and ERK (**Figure 3A**), c-fos promoter activation (**Figure 3C**), AP-1-DNA binding activation (**Figure 3D**), and c-fos nuclear translocation (**Figure 3E**) and all of these effects were markedly inhibited by magnolol (**Figure 3B–E**). Together, these results demonstrate that magnolol significantly inhibited the NF-κB and MAPK pathways and NF-κB, AP-1 nuclear translocation.

### Magnolol suppresses the development of arthritis in an AIA (adjuvant-induced arthritis) model

**Figure 4:**
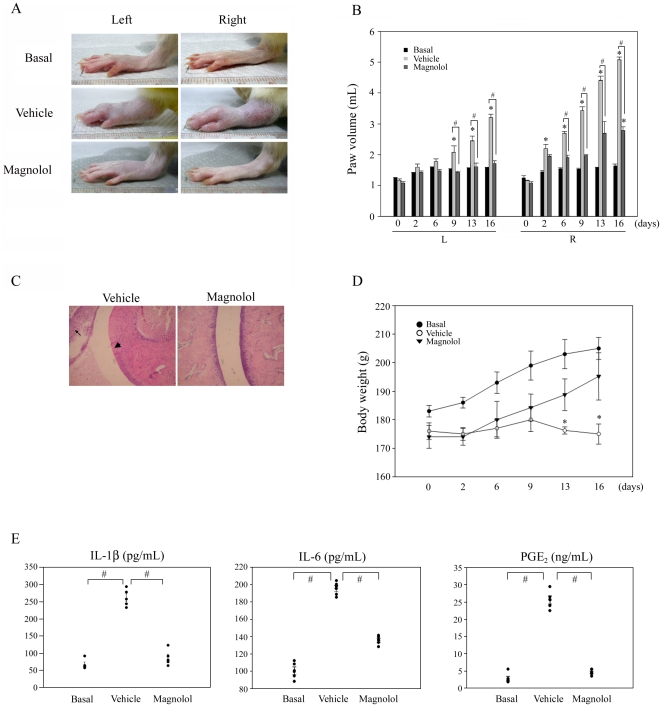
Magnolol suppresses arthritis development in an adjuvant-induced arthritis (AIA) model. After onset of arthritis, rats were treated with 100 mg/kg magnolol or vehicle (on day 2) for a total of 14 days. (A) Swelling of the ankle joints of both paws were markedly suppressed by treatment of arthritic rats with 100 mg/kg magnolol, as compared to the vehicle group. (B) Both hind paw volumes of non-treated (basal) rats, vehicle-treated, and magnolol-treated rats were measured using a digital plethysmometer on the indicated day after AIA induction. (C) Hematoxylin and eosin staining of tissue specimens from the left ankle joints of vehicle-treated rats and magnolol-treated rats. The specimen from a vehicle-treated rat exhibits severe synovitis (arrowhead) and leukocyte (arrow) infiltration. Magnolol treatment inhibited both effects. (D) Body weight of non-treated (basal) rats, vehicle-treated, and magnolol-treated rats measured on the indicated day after AIA induction. (E) Quantification of cytokines in sera by ELISA on day 16. In (B) and (D), the results are expressed as the mean ± standard error of the mean (SEM), with n = 5. * *p*<0.05 compared to the control group; #*p*<0.05 for the comparison of the indicated groups.

Our results showing that magnolol inhibited the production of inflammatory mediators through the NF-κB and MAPKs pathways strongly suggested that it might be effective in preventing the pathogenesis of destructive arthritis. We therefore examined the effect of magnolol *in vivo* by monitoring the progression and severity of an AIA model. As shown in **figure 4A–C**, compared to the vehicle-treated group, the group treated with 100 mg/kg of magnolol for 16 days not only showed significantly reduced limb swelling and paw volumes, but also markedly less leukocyte infiltration and synovitis. Treatment with magnolol did not affect the body weight loss (**Figure 4D**), suggesting that magnolol did not cause a toxic response. Furthermore, ELISA assays showed that magnolol caused a significant decrease in serum levels of IL-1β, IL-6, and PGE_2_ (**Figure 4E**). These findings suggest that magnolol has potent anti-arthritic effects *in vivo*.

## Discussion

Natural products have proved to be a valuable source of new therapeutic agents. A number of *in vitro* and *in vivo* studies have shown that magnolol has anti-inflammatory activities by inhibiting inflammatory mediator secretion [16]–[20] and suppressing inflammatory pain [23]. Another component of *Magnolia officinalis*, honokiol, was recently reported to inhibit the type II collagen-induced increase in pro-inflammatory cytokine and MMPs levels and to suppress type II collagen-induced arthritis [24], raising the possibility that magnolol may also be useful in the treatment of inflammatory arthritis. In the present study, magnolol not only significantly inhibited the IL-1β-induced increase in IL-6, PGE_2_, MMP, and COX-2 protein levels, but also remarkably alleviated *M. butyricum*-induced arthritis *in vivo* by inhibiting the NF-κB and AP-1 signaling pathways, suggesting its potential therapeutic use as a novel topical anti-arthritic agent. Previous report has indicated magnolol with low toxicity [8]; however, there was reported that high concentration of magnolol (10 µg/mL) pretreatment at 30 min before cold preservation (4°C) induces *in vitro* hepatotoxicity in rat hepatocyte clone-9 cell line under serum-reduced conditions [25]. Since low temperature may slow down or completely discontinue translational and transcriptional machineries in cold preservation cells, so that de novo synthesis (e.g. magnolol-mediated anti-apoptotic proteins (Bcl-xL) up-regulation [26]) could not be expected. Furthermore, this experiment condition was different with our study. In normothermic conditions, the same group also reported that magnolol effectively improved hepatic function and hepatocyte viability from warm ischemia-reperfusion injury in rats [26]. In addition, although PGE_2_ levels were down-regulated in response to administration of magnolol, recent study indicated that it is difficult to attribute the GI damage to one factor, PGs inhibition [27]. Further research is necessary to demonstrate the GI effect of magnolol.

IL-1β, the major mediator involved in inflammatory arthritis, can stimulate fibroblast proliferation and increase production of cytokines and enzymes, which, in turn, activate macrophages and lead to continued cytokine production [2]–[4]. This creates a positive feedback mechanism between the FLS and mononuclear cells that aggravates synovial inflammation and results in joint destruction. Thus, targeting the intracellular pathways between activated cytokine receptors and gene expression might be an attractive strategy for the treatment of inflammatory arthritis, since different pro-inflammatory mediators can share the same signaling pathway [2]. The two principal pathways activated by IL-1 are the NF-κB and MAPK pathways and the roles of both in the pathogenesis of destructive arthritis have been studied [2]–[4]. In most nonstimulated cells, NF-κB exists as an NF-κB/IκB complex in the cytoplasm; but, in response to the activation of pro-inflammatory cytokines, IκB is phosphorylated by IκB kinases and this results in free NF-κB, which can translocate into the nucleus and induce the transcription of inflammatory cytokines and mediators [3], [4]. A recent study using an adenovirus vector encoding IκBα showed that overexpression of IκBα inhibits the production of pro-inflammatory cytokines, MMPs, and aggrecanases [28], while another group demonstrated that mice lacking functional NF-κB-inducing kinase are resistant to AIA [29]. Furthermore, a potent NF-κB inhibitor, curcumin, which is derived from the dietary turmeric and forms a curcumin-phosphatidylcholine complex, is under clinical trial evaluation in osteoarthritis patients [30]. Another major transcription factor that contributes to the pathogenesis of arthritis is c-fos/AP-1, since c-fos/AP-1 not only directly controls the expression of inflammatory cytokines and MMPs by binding to AP-1 motifs in the promoters of these genes [31], but participates in a cross-talk with IL-1β to influence each other's gene expression and activity [32], [33]. These results suggest the IL-1-stimulated NF-κB and MAPK signaling pathways as potential therapeutic targets in inflammatory arthritis. Magnolol was found to suppress TNF-α-induced activation of NF-κB and AP-1, two transcription factors that regulate gene expression in human monocytes [19] and in vascular smooth muscle cells [34]. Our results showed that magnolol significantly inhibited the IL-1β-induced increase in cytokine and MMP expression and markedly inhibited the IL-1β-induced phosphorylation of IKK/IκB/p65, MAPKs and p65, c-fos DNA-binding activity and nuclear translocation; suggesting that magnolol exerts its potent anti-inflammatory activity through its dual inhibitory effects on cytokines and inflammatory mediators by regulating the NF-κB and MAPKs pathways.

We also used an AIA model to evaluate the *in vivo* anti-arthritic effect of magnolol. Injection of rats with killed *M. butyricum* suspended in complete Freund's adjuvant induces autoimmune process [35] and increases inflammatory mediators, including IL-1β, overexpression [36]. In our study, marked swelling of the right (injected) paw was seen on day 2 and of the left paw on day 9, then swelling of both paws gradually increased up to the end of the experiment on day 16. Leukocyte infiltration, synovitis, and elevated serum cytokine levels were observed in the vehicle-treated group and were much improved by administration of magnolol (100 mg/kg). Previous study observed similar result that magnolol inhibits Mac-1 (CD11b/CD18)-dependent neutrophil adhesion [37], suggest magnolol inhibits leukocyte infiltration through suppressing adhesion molecules expression.

Our observations provide evidence that magnolol exerts anti-arthritic effects by inhibiting the expression of the IL-1β-stimulated IL-6, COX-2, MMP-1, and MMP-13 inflammation-associated genes by suppressing the NF-κB and MAPKs pathways. This inhibitory effect results in an improvement in arthritic symptoms *in vivo*. Since inflammatory cytokines and MMPs play important roles in destructive arthritic disease, this suggests that magnolol might be a potential anti-arthritic agent.

## Materials and Methods

### Materials

**Figure 5:**
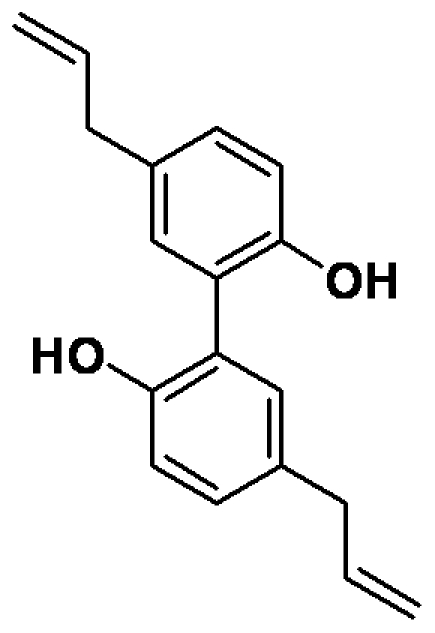
Chemical structure of magnolol.

Magnolol, with a purity greater than 98.65%, was purchased from Hanhong Chemical CO., Ltd. (Shanghai, China). Its structure is shown in **Figure 5**. Rabbit monoclonal antibodies against COX-2, IKKα, IκBα, and JNK1 were purchased from Epitomics Inc. (Burlingame, CA, USA). Rabbit polyclonal antibodies against phosphor-IKKα (Ser180)/IKKβ (Ser181), phosphor-ERK1/2 (Thr202/Tyr204), phosphor-p38 (Thr180/Tyr182), ERK1/2, c-fos, and rabbit monoclonal antibodies against phosphor-IκBα (Ser32), phosphor-p65 (Ser536), and phosphor-JNK (Thr183/Tyr185) were purchased from Cell Signaling Technology (Danvers, MA, USA). Mouse monoclonal anti-NF-κB p65 antibody was obtained from BioVision (Mountain View, CA, USA). Horseradish peroxidase (HRP)- or fluorescein isothiocyanate (FITC)- conjugated goat anti-mouse or anti-rabbit IgG antibodies were obtained from Jackson ImmunoResearch Inc. (West Grove, PA, USA). ELISA kits for human IL-6, MMP-1, and MMP-13 and for mouse IL-1β and IL-6 and a prostaglandin E_2_ immunoassay kit were purchased from R&D Systems (Minneapolis, MN, USA). The pGL4.32[*luc2P/*NF-κB-RE*/*Hygro] and p5xATF6-GL3 vectors were obtained from Promega Corp. (Madison, WI, USA) and Addgene Inc. (Cambridge, MA, USA), respectively. TurboFect™ *in vitro* transfection reagent was purchased from Fermentas (Burlington, Ontario, Canada). NF-κB and AP-1 EMSA kits were purchased from Affymetrix, Inc. (Fremont, CA, USA). All other chemicals were purchased from Sigma-Aldrich (St. Louis, MO, USA).

### Cell culture

Human FLS were derived from the synovial tissues of patients with osteoarthritis undergoing total joint replacement surgery after approval by the Ethics Committee of National Taiwan University Hospital (IRB number: 201106100RC), and the patients gave their written informed consent. The cells were cultured in 100 mm dishes in high glucose DMEM medium supplemented with 10% (v/v) heat-inactivated fetal bovine serum (FBS) (both from Invitrogen™ Life Technologies, Carlsbad, CA, USA), 100 U/mL of penicillin, and 100 µg/mL of streptomycin (Biological Industries, Kibbutz Beit Haemek, Israel) at 37°C in a humidified atmosphere of 5% CO_2_ in air. Cells from passages four to eight were used for the experiments.

### Cell viability assays

Cells (1×10^4^) in 100 µL of medium in 96-well plates were incubated with vehicle or test compound for 48 h, then 25 µL of a 1 mg/mL solution of 3-(4,5-dimethylthiazol-2-yl)-2,5-diphenyl tetrazolium bromide (MTT) was added and the mixture incubated at 37°C for 2 h. The cells were then pelleted and lysed in 100 µL of dimethyl sulfoxide and the absorbance at 550 nm measured on a microplate reader.

### RT-PCR analysis

Total RNA was isolated from cells using TRIzol reagent (Invitrogen). Single-strand cDNA for a PCR template was synthesized from 5 µg of total RNA using random primers and Moloney murine leukemia virus reverse transcriptase (Promega). The oligonucleotide primers used for the amplification were: for human IL-6 (GenBank Accession No. M14584), sense (519–540) 5′-GTT CCT GCA GAA AAA GGC AAA G-3′ and antisense (695–716) 5′-CTG AGG TGC CCA TGC TAC ATT T-3′, with a product of 198 bp; for human COX-2 (GenBank Accession No. M90100), sense (574–600) 5′-TTC AAA TGA GAT TGT GGG AAA ATT GCT-3′ and antisense (855–878) 5′-AGA TCA TCT CTG CCT GAG TAT CTT-3′, with a product of 305 bp; for human MMP-1 (GenBank Accession No. BC013875), sense (754–773) 5′-CCT AGC TAC ACC TTC AGT GG-3′ and antisense (1072–1091) 5′-GCC CAG TAC TTA TTC CCT TT-3′, with a product of 338 bp; for human MMP-13 (GenBank Accession No. BC067523), sense (1171–1190) 5′-TTG AGG ATA CAG GCA AGA CT-3′ and antisense (1462–1481) 5′-TGG AAG TAT TAC CCC AAA TG-3′, with a product of 311 bp. β-actin was used as the internal control; the β-actin primers were sense (613–632) 5′-GAC TAC CTC ATG AAG ATC CT-3′ and antisense (1103–1122) 5′-CCA CAT CTG CTG GAA GGT GG-3′, with a product of 510 bp. Equal amounts (1 µg) of each reverse-transcription product were PCR-amplified using *Taq* polymerase and 35 cycles of 1 min at 95°C, 1 min at 58°C, and 1 min at 72°C. The amplified cDNA was run on 1% agarose gels and visualized under UV light following staining with SYBR Safe DNA gel stain (Invitrogen).

### ELISA assay

FLS (1×10^6^ cells) were treated with various concentrations of magnolol for 30 min prior to stimulation with 10 ng/mL of IL-1β for 24 h in the continued presence of magnolol, then the medium was collected and assayed for IL-6, PGE_2_, MMP-1, and MMP-13 using commercial ELISA kits (R&D Systems, Minneapolis, MN, USA).

### Immunoblot analysis

Cells **(**1×10^6^) were incubated for 10 min at 4°C in lysis buffer (20 mM HEPES, pH 7.4, 2 mM EGTA, 50 mM β-glycerophosphate, 0.1% Triton X-100, 10% glycerol, 1 mM DTT, 1 µg/mL of leupeptin, 5 µg/mL of aprotinin, 1 mM phenylmethylsulfonyl fluoride, and 1 mM sodium orthovanadate), then were scraped off, incubated on ice for a further 10 min, and centrifuged at 17,000 g for 30 min at 4°C. The supernatants (60 µg of protein) were electrophoresed on SDS-PAGE and blotted onto nitrocellulose membranes. Immunoblot detection was performed with the corresponding HRP-conjugated antibodies using an ECL detection kit and exposure to photographic film.

### Transfection and reporter gene assay

5×10^4^ cells in 1 mL of growth medium were seeded in each well of 24-well plates one day before transfection. Following the manufacturer's protocol, 1 µg of plasmid pGL4.32[*luc2P/*NF-κB-RE*/*Hygro] or p5xATF6-GL3, which contains the c-fos promoter, and 1 µL of TurboFect™ transfection reagent were mixed for 20 min at room temperature, then were added to the cells and the mixtures incubated for 24 h at 37°C in a humidified atmosphere of 5% CO_2_ in air. Transfection efficiency, determined by fluorescence microscopy, was >60% in all experiments. For the reporter gene assay, 100 µL of reporter lysis buffer (Promega) was added to each well, then the cells were scraped off the dishes, centrifuged at 17,000 g for 30 s at 4°C, and the supernatants collected. Aliquots of cell lysates (20 µL) were placed in the wells of an opaque black 96-well microtiter plate and 40 µL of luciferase substrate (Promega) added and the luminescence immediately measured in a microplate luminometer (Beckman Coulter, Krefeld, Germany).

### Electrophoretic mobility shift assays (EMSAs)

Nuclear extracts were prepared and analyzed for DNA-binding activity of NF-κB and AP-1 using an EMSA Gel Shift Kit (Affymetrix) as described previously [38]. All procedures were performed according to the manufacturer's instructions.

### Confocal microscopy assay

Cells on coverslips (1×10^4^ cells) were fixed in 4% paraformaldehyde in phosphate-buffered saline (PBS) for 30 min at room temperature, then permeabilized with 0.1% Triton X-100 in PBS for 15 min on ice. Non-specific binding sites were blocked by incubation with 5% BSA in PBS overnight at 4°C. The coverslips were then incubated with primary antibodies (1∶100) in 0.5% BSA for 60 min at room temperature. After 3×10 min washes in PBS, the cells were stained for 60 min at room temperature with FITC-conjugated goat anti-mouse or anti-rabbit IgG antibodies (1∶250) in 0.5% BSA. Stained cultures were viewed and photographed under a Leica TCS SP5 confocal laser-scanning microscope using appropriate fluorescence filters.

### *In vivo* adjuvant-induced arthritis (AIA) model

Animal experiments were approved by the Institutional Animal Care and Use Committee of National Taiwan University College of Medicine (IACUC number: 20100257). Five-week-old female Lewis rats were obtained from the National Laboratory Animal Center (Taipei, Taiwan). Freund's complete adjuvant (CFA) was prepared by suspending heat-killed *Mycobacterium butyricum* (Difco) in mineral oil at 3 mg/mL. CFA-induced arthritis was induced by injection of 100 µL of the CFA emulsion intradermally into the base of the right hind paw on day 0. Magnolol [100 mg/kg solution in a vehicle mixture of 1% DMSO, 4% ethanol, 5% cremophor, and 90% of a 5% (w/v) solution of glucose in water] or vehicle was injected intraperitoneally daily from day 2 to day 16 (5 rats per group). On days 0, 2, 6, 9, 13 and 16, the animals were weighed and both paw volumes measured using a digital plethysmometer (Diagnostic & Research Instruments CO., Ltd, Taipei, Taiwan). On day 16, the rats were sacrificed and underwent histopathological assessments by hematoxylin and eosin staining of specimens from the left ankle joint.

### Data analysis

The data were expressed as the mean ± standard error of the mean (SEM) and were analyzed statistically using one-way ANOVA. When ANOVA showed significant differences between groups, Tukey post hoc test was used to determine the specific pairs of groups showing statistically significant differences. A *p* value of less than 0.05 was considered statistically significant.

## References

[1] Cooles FA, Isaacs JD (2011). Pathophysiology of rheumatoid arthritis.. Curr Opin Rheumatol.

[2] Kapoor M, Martel-Pelletier J, Lajeunesse D, Pelletier JP, Fahmi H (2011). Role of proinflammatory cytokines in the pathophysiology of osteoarthritis.. Nat Rev Rheumatol.

[3] Smolen JS, Steiner G (2003). Therapeutic strategies for rheumatoid arthritis.. Nat Rev Drug Discov.

[4] Scott DL, Wolfe F, Huizinga TWJ (2010). Rheumatoid arthritis.. Lancet.

[5] Boileau C, Martel-Pelletier J, Caron J, Msika P, Guillou GB (2009). Protective effects of total fraction of avocado/soybean unsaponifiables on the structural changes in experimental dog osteoarthritis: inhibition of nitric oxide synthase and matrix metalloproteinase-13.. Arthritis Res Ther.

[6] Gaby AR (1999). Alternative treatments for rheumatoid arthritis.. Altern Med Rev.

[7] Jacobs JWG, Kraaimaat FW, Bijlsma JW (2001). Why do patients with rheumatoid arthritis use alternative treatments?. Clin Rheumatol.

[8] Lee YJ, Lee YM, Lee CK, Jung JK, Han SB (2011). Therapeutic applications of compounds in the *Magnolia* family.. Pharmacol Ther.

[9] Seventy-third meeting of the Joint FAO/WHO Expert Committee on Food Additive (JECFA) (2011). Safety evaluation of certain food additives and contaminants.. WHO Food Additives Series.

[10] Wang JP, Ho TF, Chang LC, Chen CC (1995). Anti-inflammatory effect of magnolol, isolated from Magnolia officinalis, on A23187-induced pleurisy in mice.. J Pharm Pharmacol.

[11] Teng CM, Ko FN, Wang JP, Lin CN, Wu TS (1991). Antihaemostatic and antithrombotic effect of some antiplatelet agents isolated from Chinese herbs.. J Pharm Pharmacol.

[12] Hwang ES, Park KK (2010). Magnolol suppresses metastasis via inhibition of invasion, migration, and matrix metalloproteinase-2/-9 activities in PC-3 human prostate carcinoma cells.. Biosci Biotechnol Biochem.

[13] Chen LC, Liu YC, Liang YC, Ho YS, Lee WS (2009). Magnolol inhibits human glioblastoma cell proliferation through upregulation of p21/Cip1.. J Agric Food Chem.

[14] Teng CM, Yu SM, Chen CC, Huang YL, Huang TF (1990). EDRF-release and Ca+(+)-channel blockade by magnolol, an antiplatelet agent isolated from Chinese herb Magnolia officinalis, in rat thoracic aorta.. Life Sci.

[15] Tsai YC, Cheng PY, Kung CW, Peng YJ, Ke TH (2010). Beneficial effects of magnolol in a rodent model of endotoxin shock.. Eur J Pharmacol.

[16] Matsuda H, Kageura T, Oda M, Morikawa T, Sakamoto Y (2001). Effects of constituents from the bark of Magnolia obovata on nitric oxide production in lipopolysaccharide-activated macrophages.. Chem Pharm Bull.

[17] Schuhly W, Khan SI, Fischer NH (2009). Neolignans from North American Magnolia species with cyclooxygenase 2 inhibitory activity.. *Inflammopharmacology*.

[18] Chen YH, Lin SJ, Chen JW, Ku HH, Chen YL (2002). Magnolol attenuates VCAM-1 expression in vitro in TNF-α-treated human aortic endothelial cells and in vivo in the aorta of cholesterol-fed rabbits.. Br J Pharmacol.

[19] Tse AK, Wan CK, Zhu GY, SHen XL, Cheung HY (2007). Magnolol suppresses NF-κB activation and NF-κB regulated gene expression through inhibition of IκB kinase activation.. Mol Immunol.

[20] Park J, Lee J, Jung E, Park Y, Kim K (2004). In vitro antibacterial and anti-inflammatory effects of honokiol and magnolol against Propionibacterium sp.. Eur J Pharmacol.

[21] Barksby HE, Lea SR, Preshaw PM, Taylor JJ (2007). The expanding family of interleukin-1 cytokines and their role in destructive inflammatory disorders.. Clin Exp Immunol.

[22] Daheshia M, Yao JQ (2008). The interleukin 1β pathway in the pathogenesis of osteoarthritis.. J Rheumatol.

[23] Lin YR, Chen HH, Lin YC, Ko CH, Chan MH (2009). Antinociceptive actions of honokiol and magnolol on glutamatergic and inflammatory pain.. J Biomed Sci.

[24] Kim KR, Park KK, Chun KS, Chung WY (2010). Honokiol inhibits the progression of collagen-induced arthritis by reducing levels of pro-inflammatory cytokines and matrix metalloproteinases and blocking oxidative tissue damage.. J Pharmacol Sci.

[25] Kao YH, Jawan B, Sun CK, Goto S, Lin YC (2010). High concentration of magnolol induces hepatotoxicity under serum-reduced conditions.. Phytomedicine.

[26] Jawan B, Goto S, Pan TL, Lai CY, Luk HN (2003). The protective mechanism of magnolol, a Chinese herb drug, against warm ischemia-reperfusion injury of rat liver.. J Surg Res.

[27] Suleyman H, Albayrak A, Bilici M, Cadirci E, Halici Z (2010). Different mechanisms in information and prevention of indomethacin-induced gastric ulcers.. Inflammation.

[28] Feldmann M, Andreakos E, Smith C, Bondeson J, Yoshimura S (2002). Is NF-κB a useful therapeutic target in rheumatoid arthritis?. Ann Rheum Dis.

[29] Aya K, Alhawagri M, Hagen-Stapleton A, Kitaura H, Kanagawa O (2005). NF-κB-inducing kinase controls lymphocyte and osteoclast activities in inflammatory arthritis.. J Clin Invest.

[30] Belcaro G, Cesarone MR, Dugall M, Pellegrini L, Ledda A (2010). Efficacy and safety of Meriva®, a curcumin-phosphatidylcholine complex, during extended administration in osteoarthritis patients.. Altern Med Rev.

[31] Aikawa Y, Morimoto K, Yamamoto T, Chaki H, Hashiramoto A (2008). Treatment of arthritis with a selective inhibitor of c-Fos/activator protein-1.. Nat Biotech.

[32] Whitmarsh AJ, Shore P, Sharrocks AD, Davis RJ (1995). Integration of MAPK kinase signal transcription pathways at the serum response element.. Science.

[33] Dayer JM (2003). The pivotal role of interleukin-1 in the clinical manifestations of rheumatoid arthritis.. Rheumatology.

[34] Kim HM, Bae SJ, Kim DW, Kim BK, Lee SB (2007). Inhibitory role of magnolol on proliferative capacity and matrix metalloproteinase-9 expression in TNF-α-induced vascular smooth muscle cells.. Int Immunopharmacol.

[35] Holoshitz J, Naparstek Y, Ben-Nun A, Cohen IR (1983). Lines of T lymphocytes induce or vaccinate against autoimmune arthritis.. Science.

[36] Barsante MM, Roffe E, Yokoro CM, Tafuri WL, Souza DG (2005). Anti-inflammatory and analgesic effects of atorvastatin in a rat model of adjuvant-induced arthritis.. Eur J Pharmacol.

[37] Shen YC, Sung YK, Chen CF (1998). Magnolol inhibits Mac-1 (CD11b/CD18)-dependent neutrophil adhesion: relationship with its antioxidant effect.. Eur J Pharmacol.

[38] Chen PH, Yang CR (2008). Decoy receptor 3 expression in AsPC-1 human pancreatic adenocarcinoma cells via the phosphatidylinositol 3-kinase-, Akt-, and NF-κB-dependent pathway.. J Immunol.

